# COVID-19 Infection Complicated by a Complete Occlusion of the Left Circumflex Artery With Acute Restenosis After Drug-Eluting Stent Placement

**DOI:** 10.7759/cureus.10708

**Published:** 2020-09-29

**Authors:** Nathan Zaher, Yasar Sattar, Syed Mahmood, Tom Vacek, M Chadi Alraies

**Affiliations:** 1 Internal Medicine, Detroit Medical Center/Wayne State University School of Medicine, Detroit, USA; 2 Internal Medicine, Icahn School of Medicine at Mount Sinai, New York, USA; 3 Interventional Cardiology, Detroit Medical Center Sinai Grace, Detroit, USA; 4 Interventional Cardiology, Detroit Medical Center/Wayne State University School of Medicine, Detroit, USA; 5 Interventional Cardiology, Detroit Medical Center Cardiovascular Institute, Detroit, USA

**Keywords:** covid-19, acs, stemi, cardiac arrest

## Abstract

Severe acute respiratory syndrome coronavirus 2 (SARS-CoV-2) can cause a hypercoagulable state that can complicate the management of patients presenting with acute myocardial infarction (MI). We present the case of a patient with coronavirus disease 2019 (COVID-19) with ST elevation MI who was treated with percutaneous coronary intervention and stenting to the left circumflex artery. He was treated appropriately with anticoagulation with appropriate activated clotting time. However, the coronary angiogram course was complicated with heavy thrombosis that involved the left circumflex artery and the left anterior descending artery. Physicians are urged to suspect heparin resistance in COVID-19 patients, particularly if those patients have venous thromboembolism or acute coronary syndrome while taking heparin.

## Introduction

During the ongoing coronavirus disease 2019 (COVID-19), a variety of cardiovascular and thromboembolic manifestations have been reported in the literature. Cardiovascular or thromboembolic complications can include acute coronary syndrome (ACS) with new ischemic events and can occur in stent restenosis when using dual antiplatelet therapy for prolonged periods [1,2]. The incidence of venous thromboembolism (VTE) is estimated to be approximately 25% of COVID-19 patients. Interestingly, up to 8% to 10% of COVID-19 patients on anticoagulation therapy are also at risk of thrombosis. ACS can constitute 1.1% of thrombosis reports in COVID-19 patients who are on anticoagulation [3]. Thrombosis during anticoagulation therapy is either attributed to underdosing or heparin resistance secondary to COVID-19 in patients receiving therapeutic doses of heparin [4].

## Case presentation

**Figure 1:**
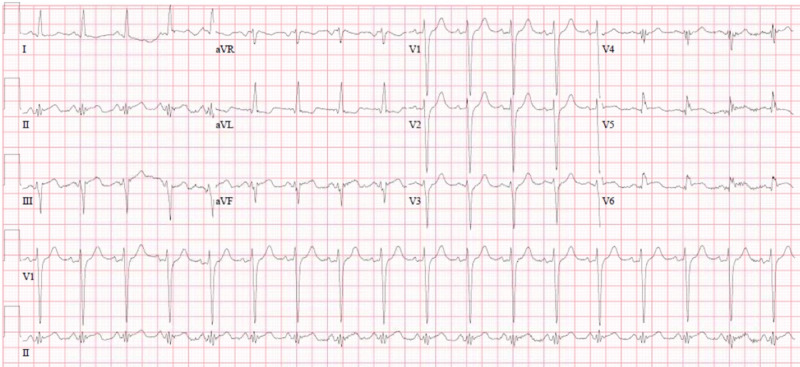
Electrocardiogram Electrocardiogram showing ischemic findings in the anterior and lateral leads

A 51-year-old man presented to the emergency department (ED) with concerns of shortness of breath and squeezing midsternal chest pain for one day. He denied any diarrhea, constipation, tobacco use, alcohol use, illicit drug use, or family history of cardiac disease. The patient’s past medical history included a previous myocardial infarction with a drug-eluting stent (DES) placed in the left anterior descending artery (LAD), coronary artery disease, hypertension, and diabetes. His medications included ticagrelor, carvedilol, atorvastatin, and aspirin. In the ED, his blood pressure was 165/112 mmHg, heart rate was 110 beats per minute, and oxygen saturation was 91% on 15 L/minute by a non-rebreather mask. Physical examination revealed the patient was experiencing anxiety and in moderate to severe distress, with tachycardia, tachypnea, bilateral rales, and increased work of breathing. The differential diagnosis included ACS, acute myocarditis, coronary vasospasm, and coronary artery dissection. Chest X-ray at presentation demonstrated diffuse B-lines and pulmonary vascular congestion. Initial electrocardiogram showed ST elevations in leads III, aVF, V5, and V6 with some reciprocal depressions in lead aVL, as well as left axis deviation and left ventricular hypertrophy (Figure 1). A bedside echocardiogram revealed a dilated left ventricle with an ejection fraction <40% and diffuse defects in LV contractility. Pertinent laboratory results revealed an elevated high sensitivity troponin I level at 175 ng/mL (normal is 3-17). A nasopharyngeal swab for severe acute respiratory syndrome coronavirus 2 reverse polymerase chain reaction (PCR) was obtained. The patient was intubated in the ED for acute hypoxic respiratory failure secondary to COVID-19 with positive PCR.

**Figure 2:**
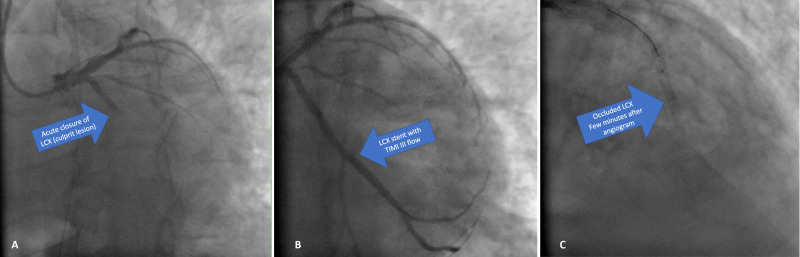
Coronary Angiogram Angiograms showing initial occlusion of the left circumflex artery (A), then thrombolysis in myocardial infarction flow with stenting (B), and then restenosis a few minutes later (C). LCX, left circumflex artery; TIMI III, thrombolysis in myocardial ischemia grade 3.

The catheterization laboratory was activated, given concerns for ACS. Before percutaneous coronary intervention (PCI), the patient received heparin with an activated clotting time (ACT) of 300 seconds. Coronary angiogram showed 100% occlusion of the left circumflex artery (LCX) with diffuse disease in the LAD (Figure 2A). Using a workhorse wire, the LCX lesion was easily crossed, and a percutaneous transluminal coronary angioplasty was performed. A 2.5 mm x 18 mm DES was deployed in the mid-LCX, and a 3.0 mm x 23 mm DES was deployed in the proximal LCX. An angiogram performed before removing the guidewire showed complete restoration of flow in the LCX artery (Figure 2B). However, after the stents were placed, the guidewire was removed, the final angiogram showed acute rethrombosis of the LCX minutes later (Figure 2C).

The guidewire was immediately reinserted across the LCX lesion. Thirty-five minutes after the initial ACT, a repeat ACT was drawn, and results were subtherapeutic at 167 seconds (down from 300 seconds). The patient was given an additional 4,000 units of heparin, and balloon angioplasty was performed for the LCX stenosis. The patient’s condition started to deteriorate after acute restenosis of the stent with subsequent sudden cardiac arrest.

## Discussion

Cardiovascular complications and thromboembolism in the setting of COVID-19 have been commonly reported, with abnormal cardiac biomarkers seen in up to 12% of COVID-19 patients. Cardiovascular damage in the setting of COVID-19 can be attributed to a prothrombotic state and/or an inflammatory state causing plaque rupture. Cardiovascular mortality can occur in up to 52% of COVID-19 patients [5]. Furthermore, COVID-19 can cause hypercoagulability. Pathophysiological mechanisms of hypercoagulation causing VTE include a cytokine storm, clotting factor, and clotting cascade activation. This can be depicted with elevated D-dimer levels [6].

The European Society of Cardiology recommended anticoagulation therapy in the setting of ACS and COVID-19 using a heparin drip with a goal of partial thromboplastin time (PTT) of 60 to 85 seconds (intensive care unit [ICU] patients) or enoxaparin 1 mg/kg twice daily (non-ICU patients) [7]. The literature mentions ongoing thrombosis or worsening thrombosis during anticoagulation therapy in the setting of COVID-19. A study by Beun et al. provided an interesting point about potential heparin resistance due to COVID-19 and suggested the use of anti-Xa levels as a marker of the therapeutic range of anticoagulation [4,8,9]. The authors determined that 33% of patients experienced VTE while receiving anticoagulation during ICU admission. A rare phenomenon called heparin resistance has been noted in some COVID-19 patients receiving more than 35,000 IU/day unfractionated heparin to achieve target PTT or failure to achieve anticoagulation while at therapeutic PTT levels [10]. Beun et al. reported normal antithrombin and high factor VIII levels in participants [4]. High factor VIII levels were presumed to cause heparin resistance. Our patient also had presumed heparin resistance with new acute onset of restenosis of the stent immediately after PCI. Restenosis has been observed in COVID-19 cases; however, none of the reports showed such acute restenosis within minutes. Another study by Prieto-Lobato et al. provided recommendations for maintaining the same antithrombotic treatment and PCI for ACS in COVID-19 patients as for COVID-19 negative patients [2].

## Conclusions

The future debate will be whether the guideline for anticoagulation therapy for ACS in the setting of COVID-19 is sufficient. It is imperative to suspect heparin resistance in COVID-19 patients, particularly if they have thrombosis while on anticoagulation. This information is vital as it could prevent unfortunate outcomes, such as what we witnessed here. 

## References

[1] Hendren NS, Drazner MH, Bozkurt B, Cooper LT Jr (2020). Description and proposed management of the acute COVID-19 cardiovascular syndrome. Circulation.

[2] Prieto-Lobato A, Ramos-Martínez R, Vallejo-Calcerrada N, Corbí-Pascual M, Córdoba-Soriano JG (2020). A case series of stent thrombosis during the COVID-19 pandemic. JACC Case Rep.

[3] Lodigiani C, Iapichino G, Carenzo L (2020). Venous and arterial thromboembolic complications in COVID-19 patients admitted to an academic hospital in Milan, Italy. Thromb Res.

[4] Beun R, Kusadasi N, Sikma M, Westerink J, Huisman A (2020). Thromboembolic events and apparent heparin resistance in patients infected with SARS-CoV-2. Int J Lab Hematol.

[5] Zhou F, Yu T, Du R (2020). Clinical course and risk factors for mortality of adult inpatients with COVID-19 in Wuhan, China: a retrospective cohort study. Lancet.

[6] Tang N, Bai H, Chen X, Gong J, Li D, Sun Z (2020). Anticoagulant treatment is associated with decreased mortality in severe coronavirus disease 2019 patients with coagulopathy. J Thromb Haemost.

[7] Atallah B, Mallah SI, Al Mahmeed W (2020). Anticoagulation in COVID-19. Eur Heart J Cardiovasc Pharmacother.

[8] Samuel S, Allison TA, Sharaf S (2016). Antifactor Xa levels vs. activated partial thromboplastin time for monitoring unfractionated heparin. A pilot study. J Clin Pharm Ther.

[9] Guervil DJ, Rosenberg AF, Winterstein AG, Harris NS, Johns TE, Zumberg MS (2011). Activated partial thromboplastin time versus antifactor Xa heparin assay in monitoring unfractionated heparin by continuous intravenous infusion. Ann Pharmacother.

[10] Downie I, Liederman Z, Thiyagarajah K, Selby R, Lin Y (2019). Pseudo heparin resistance caused by elevated factor VIII in a critically ill patient. Can J Anaesth.

